# Conditional disruption of interactions between Gα_i2_ and regulator of G protein signaling (RGS) proteins protects the heart from ischemic injury

**DOI:** 10.1186/2050-6511-15-29

**Published:** 2014-06-05

**Authors:** Sergio Parra, Xinyan Huang, Raelene A Charbeneau, Susan M Wade, Kuljeet Kaur, Boyd R Rorabaugh, Richard R Neubig

**Affiliations:** 1 Department of Pharmacology, University of Michigan, Ann Arbor, Michigan 48109, USA; 2 Department of Pharmaceutical and Biomedical Sciences, Ohio Northern University College of Pharmacy, Ada, OH 45810, USA; 3 Department of Pharmacology and Toxicology, B440 Life Sciences, Michigan State University, 1355 Bogue St, East Lansing, MI 48824, USA

**Keywords:** G protein coupled receptors, Ischemia-reperfusion, Cre-*LoxP*, Mutation, cAMP inhibition, Regulator of G protein signaling, RGS

## Abstract

**Background:**

Regulator of G protein signaling (RGS) proteins suppress G protein coupled receptor signaling by catalyzing the hydrolysis of Gα-bound guanine nucleotide triphosphate. Transgenic mice in which RGS-mediated regulation of Gα_i2_ is lost (RGS insensitive Gα_i2_^G184S^) exhibit beneficial (protection against ischemic injury) and detrimental (enhanced fibrosis) cardiac phenotypes. This mouse model has revealed the physiological significance of RGS/Gα_i2_ interactions. Previous studies of the Gα_i2_^G184S^ mutation used mice that express this mutant protein throughout their lives. Thus, it is unclear whether these phenotypes result from chronic or acute Gα_i2_^G184S^ expression. We addressed this issue by developing mice that conditionally express Gα_i2_^G184S^.

**Methods:**

Mice that conditionally express RGS insensitive Gα_i2_^G184S^ were generated using a floxed minigene strategy. Conditional expression of Gα_i2_^G184S^ was characterized by reverse transcription polymerase chain reaction and by enhancement of agonist-induced inhibition of cAMP production in isolated cardiac fibroblasts. The impact of conditional RGS insensitive Gα_i2_^G184S^ expression on ischemic injury was assessed by measuring contractile recovery and infarct sizes in isolated hearts subjected to 30 min ischemia and 2 hours reperfusion.

**Results:**

We demonstrate tamoxifen-dependent expression of Gα_i2_^G184S^, enhanced inhibition of cAMP production, and cardioprotection from ischemic injury in hearts conditionally expressing Gα_i2_^G184S^. Thus the cardioprotective phenotype previously reported in mice expressing Gα_i2_^G184S^ does not require embryonic or chronic Gα_i2_^G184S^ expression. Rather, cardioprotection occurs following acute (days rather than months) expression of Gα_i2_^G184S^.

**Conclusions:**

These data suggest that RGS proteins might provide new therapeutic targets to protect the heart from ischemic injury. We anticipate that this model will be valuable for understanding the time course (chronic versus acute) and mechanisms of other phenotypic changes that occur following disruption of interactions between Gα_i2_ and RGS proteins.

## Background

G protein coupled receptor signaling is mediated by four major families of G proteins (Gα_s_, Gα_i/o_, Gα_q_, and Gα_12_). Gα_i_ coupled receptors play important roles in regulating inflammation, cardiovascular function, endocrine signaling, drug abuse, and anxiety/depression – related behaviors [1-5]. Three different Gα_i_- isoforms (Gα_i1_, Gα_i2_, Gα_i3_) have been cloned and characterized. Gαi/o-coupled responses can be distinguished from responses mediated by other families of G proteins by their sensitivity to pertussis toxin, but the *in vivo* roles of individual Gα_i/o_ isoforms remain obscure.

Signaling through Gα_i_ is suppressed by regulator of G protein signaling (RGS) proteins which terminate Gα_i_ signaling by accelerating the hydrolysis of Gα-bound guanine nucleotide triphosphate (GTP) [6,7]. More than 20 different RGS proteins have been identified. However identifying the physiological roles of individual RGS proteins through selective pharmacological inhibition is not yet possible. Knockout mouse models in which individual RGS proteins have been genetically deleted have been useful in identifying the functions of some RGS proteins, but this approach is complicated by the potential for compensation of the lost RGS protein by other RGS proteins that may have redundant functions. Consequently, we have exploited a mutation in Gα_i2_ (Gα_i2_^G184S^) that blocks RGS binding and activity [8]. This allows us to define the functions of this individual Gα_i_-isoform and to explore the physiological roles of interactions between Gα_i2_ and endogenous RGS proteins [8,9]. Knock-in mice expressing Gα_i2_^G184S^ show enhanced receptor-dependent signaling through Gα_i2_[8,9], and they exhibit a number of interesting cardiac phenotype changes including enhanced carbachol-induced bradycardia, enhanced isoproterenol-induced cardiac fibrosis, and premature death in a dilated cardiomyopathy model [8-12]. Hearts from both homozygous and heterozygous Gα_i2_^G184S^ mutant mice also exhibit smaller infarcts and better recovery of cardiac contractile function following an ischemic insult [13]. Genetic disruption of interactions between Gα_i2_ and endogenous RGS proteins also produces several noncardiac phenotypic changes including antidepressant-like behavior [5], resistance to high fat diet-induced weight gain, decreased body fat, and protection from the development of insulin resistance [14].

One limitation of this transgenic model is that the Gα_i2_^G184S^ mutation is chronically expressed throughout the prenatal and postnatal life of the animal. Thus, it is unclear whether the phenotypes that have been reported result from changes during embryological development and whether the phenotypes require chronic or only acute disruption of interactions between Gα_i2_ and endogenous RGS proteins. In the present study, we addressed this issue by creating a conditional knock-in mouse model that conditionally expresses RGS insensitive Gα_i2_^G184S^. This study focused on the ability of acute Gα_i2_^G184S^ expression to protect the heart from ischemic injury.

## Methods

### Gene targeting

**Figure 1:**
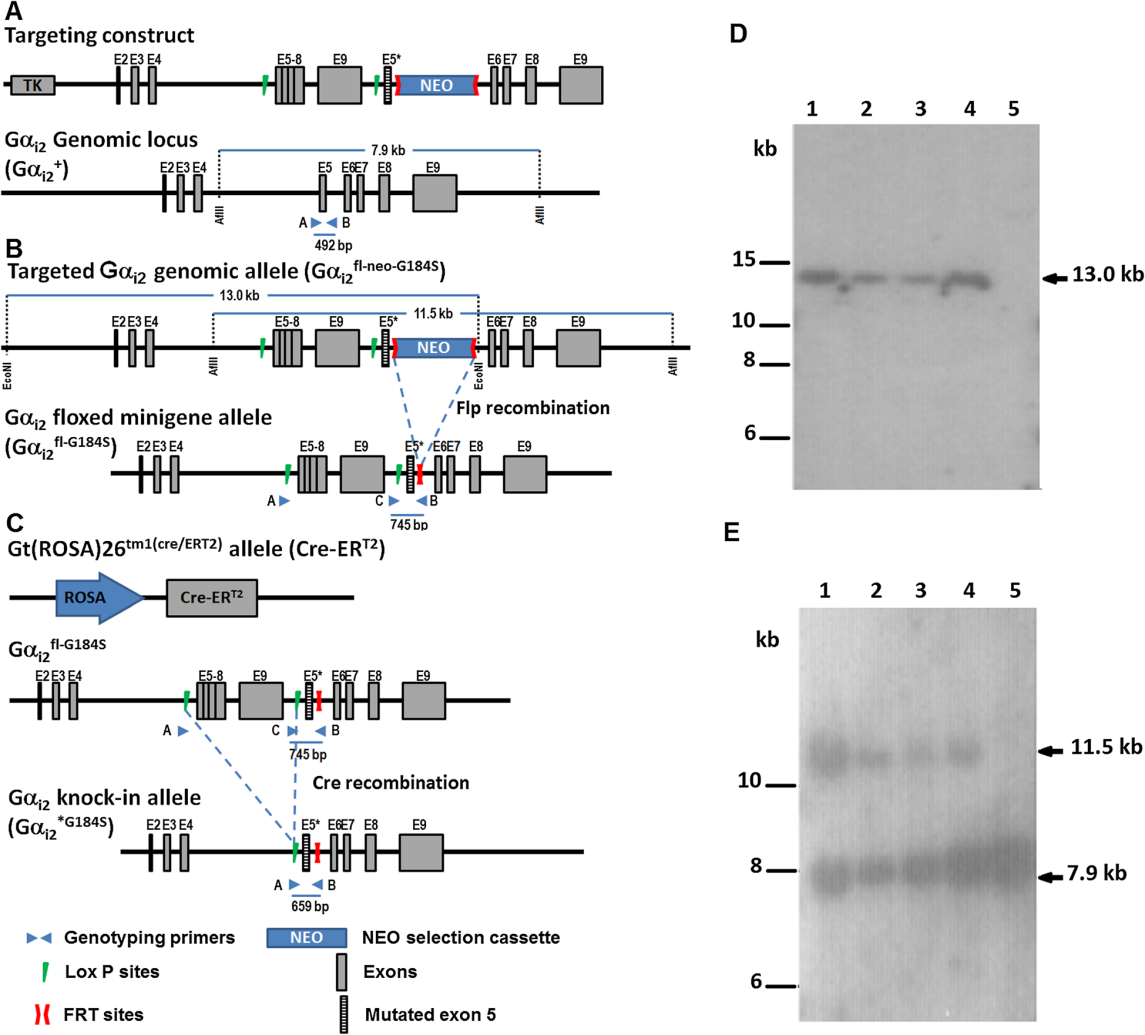
**Floxed mini-gene targeting strategy for the generation of conditional “knock-in” of Gα**_i2_^*G184S ^**allele. A** Exon 5 of the genomic locus of the gene Gnai2 was replaced by homologous recombination using a vector containing a G184S mutant Exon 5 (E5*) with a 3’ extension containing Flp Recombinase Target (FRT)-sites (red chevrons) flanking a neo selection cassette and a 5’ extension with Lox P sites (green triangles) flanking a minigene containing wild-type E5-9. **B** After introduction of the targeting vector, the neo marker is removed by breeding the Gα_i2_^fl-neo-G184S^ positive mice with transgenic Flp recombinase expressing animals. **C** To generate whole-body Gα_i2_^*G184S^ conditional knock-in mice, transgenic mice carrying tamoxifen-sensitive, Cre-ER^T2^ gene driven by the Gt(ROSA)26Sor^tm1^ (ROSA) promoter were bred with Gα_i2_^fl/fl-G184S^ mice. After tamoxifen administration there is recombination of genomic Lox P sites which leads to removal of the E5-9 minigene and the mutant E5* is positioned in the normal Gnai2 gene to express the Gα_i2_*^G184S^ mutant allele. **D** Southern blot analysis of ES cell genomic DNA following digestion with EcoNI and hybridization with the 5’-Neo probe to screen for 5’ homologous recombination event. **E** Southern blot verification analysis of ES cell genomic DNA following digestion with AflII and hybridization with an external 3’ probe. In figures *d* and *e* lanes 1 to 4 are correctly targeted ES cell clones, lane 5 is a non-targeted (WT) ES cell clone.

All protocols and procedures were approved by the University Committee on Use and Care of Animals at the University of Michigan and by the Animal Care and Use Committee of Ohio Northern University. Experimental procedures and animal husbandry were conducted in accordance with NIH guidelines. Animals were housed in a specific pathogen free facility with a 12 hour: 12 hour light: dark cycle and free access to food and water. Mice expressing RGS-insensitive Gα_i2_^G184S^ were generated by a “floxed minigene” strategy [15,16]. The targeting construct to create the conditional knock-in of the G184S mutation at Gα_i2_ (Figure 1A) was generated from a 129 BAC library in a manner similar to that previously described for the conventional Gα_i2_^G184S^ knock-in mutant mice [11]. Elements included: a 6.4 kb 5’ homology arm containing the endogenous Gnai2 sequence encompassing exons 2 to 4, a minigene version of the wild type Gnai2 gene composed of the fused exons 5 to 8, intron 8 and exon 9 (this *Gnai2* wild-type minigene was flanked with *loxP* sites allowing its deletion under the action of the Cre recombinase), the mutated exon 5 containing the G184S mutation (GGGC to TAGT mutation in position 85 in exon 5), a neo selection cassette flanked with FRT sites allowing its deletion under the Flp recombinase action, and a 2.4 kb 3’ homology arm containing the endogenous Gnai2 sequence encompassing exons 6 to 9 and sequence located downstream of the gene. In addition, AflII and SacI restriction sites were inserted in the 5’ homology arm at the BstEII site between Exon 4 and the 5’ *loxP* site to be used for Southern blot analysis of the targeted ES cells. A Thymidine Kinase (TK) negative selection marker was inserted upstream of the 5’ homology arm. ES cells (5 × 10^6^ cells - SV129 strain) were electroporated (GenOway; Lyon, France) with 40 μg of linearized plasmid and selection started 48 hours later with 200 ug/ml of G418 followed by selection in GANIX to enhance the likelihood of homologous recombination. Targeted cells were identified by 3’ genomic PCR. Of the 23 positive clones, five also showed the appropriate presence of the AflII/SacI restriction site on 5’ PCR. All five were confirmed by 5’ and 3’ Southern Blot analysis (13.0 kb EcoNI fragment and 11.5 kb AflII fragment, respectively) (GenOway; Lyon, France).

### Mouse generation

Three independent embryonic stem (ES) cell lines with normal karyotype were used for blastocyst injection into C57BL/6J host blastocysts. Highly chimeric males were bred to C57BL/6J female mice and F1 agouti offspring mice were genotyped by PCR and Southern blot hybridization to validate germline transmission (GenOway; Lyon, France). To delete the FRT-flanked neo gene, offspring showing the presence of the Gα_i2_^+/fl-neo-G184S^ allele by genomic PCR were crossed against beta-actin FLPe mice (from the University of Michigan Transgenic Animal Model Core). Litters from this cross were analyzed for the presence of the FLP transgene as well as Gα_i2_ genotypes by genomic PCR. FLP/Gα_i2_^+/fl-G184S^ mice lacking the neo cassette were backcrossed onto the C57BL/6J strain to eliminate the FLPe gene. To generate whole-body Gα_i2_^G184S^ conditional knock-in mice, transgenic mice carrying the tamoxifen-sensitive, Cre-ER^T2^ gene driven by the ROSA26 promoter (The Jackson Laboratory; Bar Harbor, ME) were bred with Gα_i2_^fl-G184S/fl-G184S^ mice. All mice used in this study have been backcrossed for five generations onto the C57BL/6J strain. They were between 8 – 20 weeks old and the number for each experiment is indicated in the figure legends. Animals were maintained in a specific pathogen-free facility with a 12-h light/dark cycle and fed standard laboratory chow and water ad libitum.

### Genotyping

Genomic DNA was isolated from mouse tail using the HotSHOT method [17] and analyzed in a single PCR reaction using two forward primers A and C (GAGCCCATGTTTCTTAAAGAAGCAAGGATA and TCCCACACCTTAGTGCCACACCT, respectively) and reverse primer B (TGAGGACATGCCTTCCCAACACAAT), which amplify the Gα_i2_^+^ (492-bp) and Gα_i2_^fl-G184S^ (745-bp) alleles, respectively.

### Tamoxifen preparation and treatment

Tamoxifen (Sigma, T5648) was prepared in peanut oil (Sigma, P2144) containing 10% ethanol at a concentration of 60 mg/ml [16]. Daily doses of 50 mg/kg over five days were given by intraperitoneal injection to induce recombination. Control mice were treated with vehicle (90% peanut oil/10% ethanol) alone.

### Blood and tissue collection

Blood was withdrawn from the saphenous vein into dipotassium ethylenediamine tetraacetic acid -coated microtubes before the first and 72 hours after the last dose of tamoxifen and then stored at -80˚C until processing for analysis. Mice were euthanized by pentobarbital injection and tissues were quickly removed, snap-frozen in liquid nitrogen, and stored at -80°C until analysis.

### Recombination assessment

Qualitative assessment for recombination, before and after tamoxifen treatment, was performed by polymerase chain reaction (PCR) using genomic DNA isolated from whole blood and tissue samples from genotyped mice carrying one copy of the ROSA Cre-ER^T2^ gene and the indicated Gα_i2_ alleles (Gα_i2_^+/+^or Gα_i2_^G184S^_)_ was amplified by using forward primer A and reverse primer B described above. This PCR reaction generates a 659-bp band only after Cre recombination. PCR reactions for testing genotype and recombination were carried out under the following conditions: 95°C for 5 min, 35 cycles at 95°C, and 64.5°C each for 30 s, followed by extension at 72°C for 1 min and final extension at 72°C for 2 min.

### Western blotting

Tissues were dissected and homogenized in a Bullet Blender homogenizer (Next Advance; Averill Park, NY) with RIPA buffer containing protease inhibitors (20 mM Tris-HCl, pH7.4, 150 mM NaCl, 1 mM EDTA, 1 mM β-glycerophospate, 1% Triton X-100, 0.1% SDS, Complete protease inhibitor cocktail (Roche; Pleasanton, CA). Protein concentrations were measured using the BCA Protein Assay kit (Thermo Fisher Scientific; Rockford, IL). Eighty micrograms of protein were separated on a denaturing 12% SDS–PAGE gel and transferred to PVDF membrane. The membranes were washed, blocked (5% milk in PBS-T), and incubated in the appropriate antibodies overnight at 4°C. Antibodies for Western blot analysis: rabbit anti-Gα_i2_ (a gift from Dr. Susanne Mumby, University of Texas Southwestern Medical Center) at a dilution of 1:1000 and mouse anti-GAPDH (Cell Signaling Technology, Danvers, MA), as loading control, at a dilution of 1:5000. Secondary antibodies were horseradish peroxidase conjugated. Visualization of the protein bands was done using the Super Signal West Pico chemiluminescent substrate (Thermo Fisher Scientific, Rockford, IL). Films were scanned and quantified using Image J software (NIH, Bethesda, MD). All samples were normalized with GAPDH as a loading control and the amount of Gα_i2_ protein in wild type tissue was set to 100%.

### Allele ratio conversion

RNA was isolated from whole blood, heart, brain, and kidney using a commercial kit (Qiagen, Valencia, CA), and reverse transcription was carried out using Reverse Transcription reagents (Applied Biosystems). PCR was performed using a set of primers that amplify a 442–base pair segment of Gα_i2_ cDNA including both wildtype and Gα_i2_^G184S^. The primers used were CCAGCGTGCGGATGATGCC and GATGAGGAGATGAACCGCATGCAT. The cDNA PCR products obtained for each sample were purified (Qiagen, Valencia, CA), and directly sequenced on an ABI 3730XL using the forward primer used for amplification of Gα_i2_ in PCR. The allelic expression ratio of mutant Gα_i2_^G184S^ to wildtype Gα_i2_ in mice was determined from the chromatogram peak heights of the bases differentiating between the Gα_i2_^G184S^ mutant and wildtype Gα_i2_ alleles. Peak heights were determined using Image J software and mutant allele expression was calculated using the formula: % mutant allele expression = (T + A + T-peak heights)/((T + A + T-peak heights) + (G + G + C-peak heights)) X 100. If any of the peaks of the mutant or the wild type sequence were not detected in the chromatogram the sample was considered 100% wild type or 100% mutant, respectively.

### Adult cardiac fibroblast culture

Adult cardiac fibroblasts (ACF) were isolated from mice as previously described [12,18]. Hearts were quickly isolated, placed in ice-cold perfusion buffer, and then perfused via an aortic cannula for 4 min with calcium-free perfusion buffer (NaCl, 113 mM; KCl, 4.7 mM; MgSO_4_-7H_2_O, 1.2 mM; Na_2_HPO_4_, 0.6 mM; KH_2_PO4, 0.6 mM; phenol red, 0.032 mM; HEPES, 10 mM; NaHCO_3_, 12 mM; KHCO_3_, 10 mM; taurine, 30 mM; glucose, 5.5 mM; and butanedione monoxime, 10 mM, pH 7.46). Hearts were then perfused for 8 min with the same buffer containing 0.5 mg/ml collagenase II (Worthington Biochemicals, Lakewood) and 1.25 μM CaCl_2_. Hearts were removed from the apparatus and the ventricles were suspended in perfusion buffer with collagenase and 1.25 μM CaCl_2_. The tissue was disrupted with fine forceps and by gentle pipetting. Cells were transferred to buffer containing 1.25 μM CaCl2 and 10% serum to stop the digestion. Samples were centrifuged at 180×g for 1 min to remove debris and myocytes. The remaining suspended fibroblasts were centrifuged at 300 × g for 10 min, then resuspended in phosphate buffered saline, centrifuged again, the supernatant was discarded, and the final cell pellet was resuspended in Dulbecco's modified Eagle’s medium supplemented with 1% penicillin/streptomycin, insulin/transferrin/selenium (ITS from Sigma) 1X, and 10% fetal bovine serum.

### cAMP production in adult cardiac fibroblasts

The effect of lysophosphatidic acid (LPA) on 3´,5´ cyclic adenosine monophosphate (cAMP) production in ACF was determined in 384-well plates using the Lance Ultra cAMP kit (PerkinElmer; Waltham, MA). Briefly, ACF (passage 2) were added at 1,000 cells per well and cAMP accumulation was initiated by adding stimulation buffer containing 0.5 mM isobutyl-1-methylxanthine and 10 μM forskolin with the indicated concentrations of LPA. After 30 min at room temperature, the reaction was stopped by lysing the cells using the buffer supplied with the PerkinElmer kit. Time-resolved fluorescence was measured with a Synergy 2 (BioTek; Winooski, VT) microplate reader.

### Langendorff isolated heart preparation and infarct size measurement

Mice were treated with tamoxifen or vehicle for 5 days as described above. Fourteen days after the first injection, mice were anesthetized and heparinized by a single injection containing pentobarbital (100 mg/kg) and heparin (150 mg/kg). Hearts were immediately excised and cannulated while bathed in ice-cold Krebs solution. Krebs solution was perfused through the aortic cannula at a constant pressure of 75 mmHg. Hearts were subjected to 30 minutes of ischemia and 2 hours reperfusion as previously described [13]. Contractile function of the left ventricle was continuously measured using an intraventricular balloon and infarct size was measured by triphenyltetrazolium chloride staining as described previously [13].

### Statistical analyses

Data were analyzed using one-way or two-way analysis of variance, followed by a Bonferroni’s post test or a Tukey’s post test when appropriate. GraphPad Prism version 4.0 (GraphPad Software, San Diego, CA) was used for statistical analysis.

## Results

**Figure 2:**
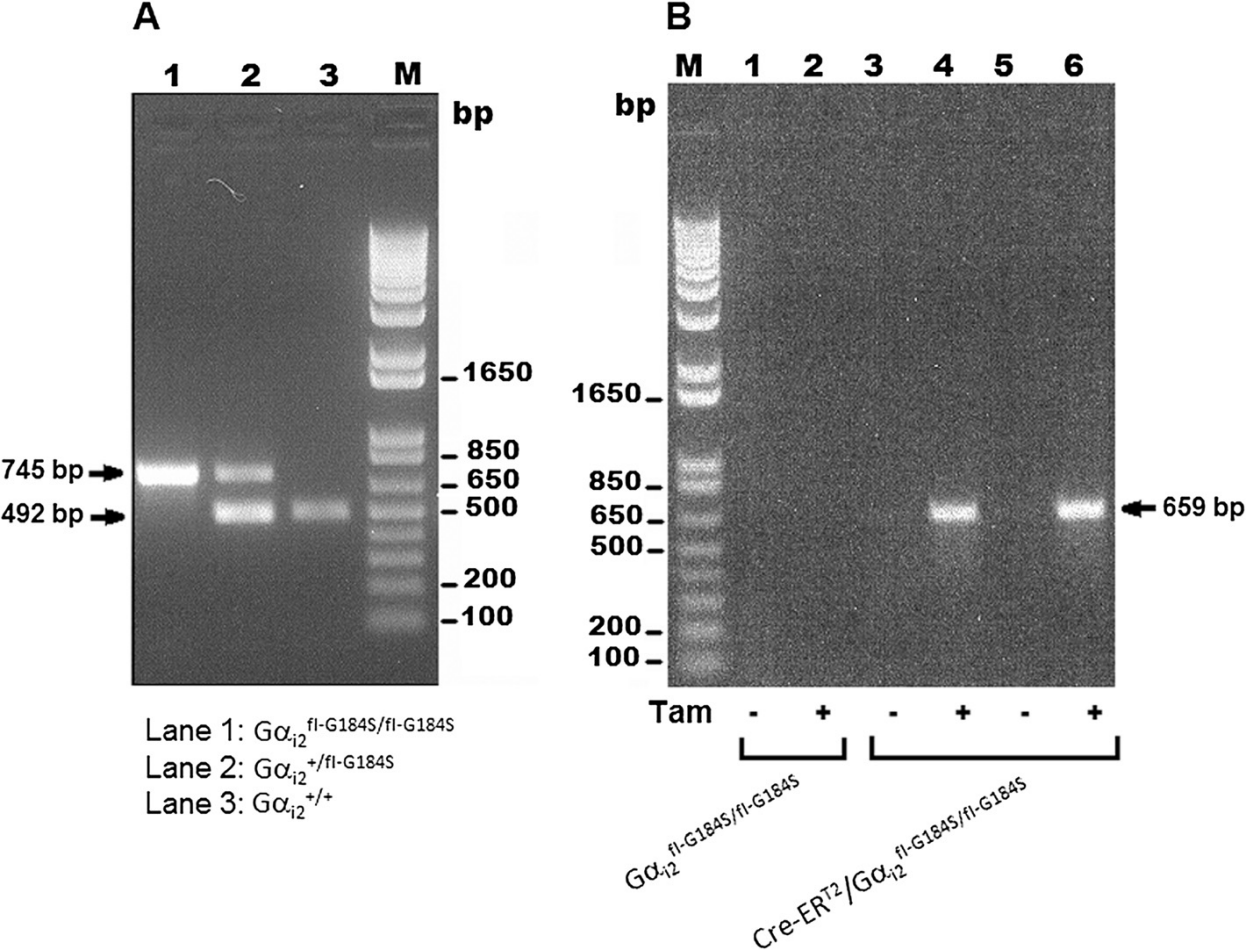
**Representative agarose gel images assessing genotypes and recombination in mice. A** PCR genotyping was done simultaneously using three primers A, B, and C from Figure 1. A 492-bp band represents the wild-type Gnai2 allele and the 745-bp band represents the Gnai2 fl-G184S conditional allele after removal of the *neo* cassette. **B** Genomic DNA extracted from blood was amplified before and after tamoxifen treatment. Lanes 1 and 2 were from Cre negative Gα ^fl-G184S/fl-G184S^ mice while lanes 3-6 were from Cre-ER^T2^, Gα_i2_^fl-G184S/fl-G184S^ mice. Tamoxifen-induced recombination was evaluated using primers A and B and confirms the in vivo conversion to the *G184S allele for expression of the RGS-insensitive Gα_i2_. The 659-bp band was exclusively generated after tamoxifen treatment (Tam). The non-recombined fl-G184S allele is not detected due to the large size of the PCR product (3189-bp).

To implement a conditional knock-in strategy, a replacement-type minigene targeting vector was used to modify the Gnai2 gene as depicted in Figure 1A-C. This construct was designed to (1) flank an exon 5 through 9 minigene with *loxP* recombination recognition sites, (2) insert a neomycin selection cassette that was flanked by FRT recombination sites downstream of the floxed wild type Gα_i2_ coding and regulatory sequences, and (3) replace the wildtype genomic exon 5 with the G184S mutant version followed by the remainder of the wildtype Gα_i2_ sequence in the normal genomic context. Because of size constraints on the cloning vector used to create the targeting construct, exons 5 through 8 were fused and introns 5-7 were deleted. Intron 8 and non-coding exon 9 were retained due to the presence of regulatory elements. The knock-in mutation was introduced into the genomic exon 5 to change the codon for glycine 184 to serine (Figure 1A). Following electroporation of the targeting construct into embryonic stem cells, G418-resistant ES cell clones were screened for gene targeting by PCR and confirmed by Southern blot analysis. Blots were hybridized to 5’ and 3’ probes. After genomic DNA digestion with EcoNI, the 5’ probe hybridized with a 13.0 kb fragment generated from a recombinant Ga_i2_^G184S^ allele (Figure 1D). This was confirmed by blotting with a 3’ probe after genomic DNA digestion with AflII. This probe hybridized to a 7.9 kb fragment from the wild type Ga^i2^ allele and an 11.5 kb fragment from a Ga_i2_^G184S^ allele (Figure 1E). One ES cell clone was not targeted (lane 5) and four clones were correctly targeted (lanes 1 to 4). Three targeted ES cell clones with normal karyotype were used to produce germline-competent chimeric mice using standard techniques. After FLP recombinase crosses, mice that were heterozygous for the targeted locus without the neomycin cassette (^fl-G184S^ allele) were subsequently backcrossed with C57BL/6J mice to eliminate the beta-actin FLP transgene. Figure 2A shows representative results from the PCR screening used to select mice with the desired genotype.

To demonstrate a global, time-dependent, and Cre-mediated conversion of the conditional allele (^fl-G184S^) to the RGS insensitive allele (^G184S^), we crossed mice that were homozygous for the fl-G184S allele (Gα_i2_^fl-G184S/fl-G184S^) mice with a tamoxifen-dependent Cre general deleter mouse line [19-21]. This line expresses a tamoxifen-sensitive Cre (Cre-ER^T2^) from the ROSA26 locus (Figure 1C). When tamoxifen is administered, its metabolite 4-OH tamoxifen binds to the Cre-ER^T2^ which translocates to the nucleus facilitating recombination of genomic *loxP* sites. As shown in Figure 2B, this removes the E5-9 minigene and positions mutant Exon 5 (E5*) in the normal Gnai2 genomic context to express the mutant Gα_i2_^G184S^ protein. Gα_i2_^fl-G184S/fl-G184S^ and Cre-ER^T2^/Gα_i2_^fl-G184S/fl-G184S^ mice are viable and do not show any gross developmental abnormalities.

**Figure 3:**
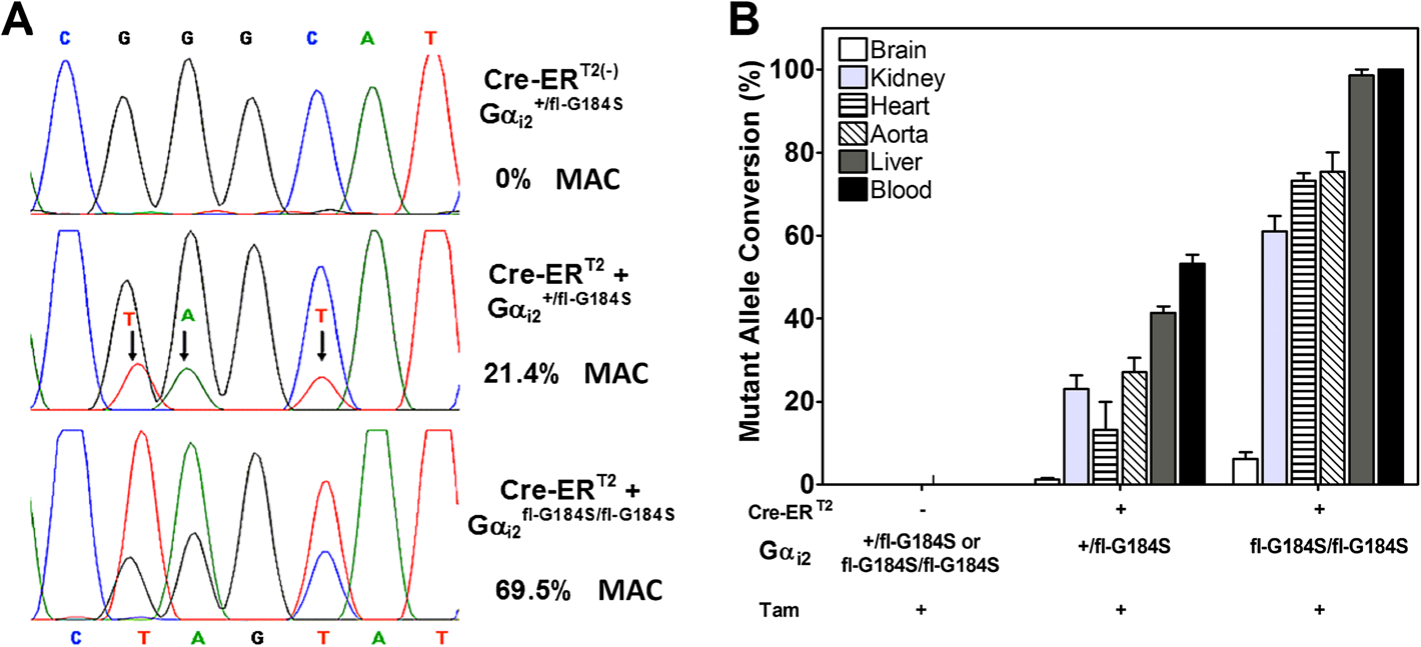
**Efficiency and genotype specificity of Cre-induced mutant allele mRNA conversion (MAC) in tissues after tamoxifen treatment in Gα**_**i2**_^**fl-G184S **^**mice. A** Representative chromatograms of sequenced PCR products from mRNA purified from hearts of tamoxifen-treated mice (50 mg/kg i.p. on five consecutive days). Mice lacking Cre (Cre-ER^T2-^) showed no evidence of the TAGT sequence expected for G184S mutant mRNA (top chromatogram). For mice expressing Cre (Cre-ER^T2+^), the percentage conversion to the mutated sequence (% MAC) was higher in mice that were homozygous for the G184S mutation (Gα_i2_^fl-G184S/fl-G184S^, lower chromatogram) than in heterozygous (Gα_i2_^+/fl-G184S^, middle chromatogram) animals. **B** The extent of recombination was higher in blood and liver than in other tissues with heart showing approximately 80% MAC in homozygotes. Values are expressed as the mean (±SE, n = 3) of the percentage of the mutated allele determined from the added peak heights of the three changed bases (see Methods).

Reverse transcription-polymerase chain reaction (RT-PCR) analysis was used to amplify a 442-bp cDNA product expressed from the Gnai2 gene to assess the ratio of mutant to wildtype Gα_i2_ RNA expression. Only the Cre-ER^T2^/Gα_i2_^+/fl-G184S^, or Cre-ER^T2^/Gα_i2_^fl-G184S/fl-G184S^ mice express mutant mRNA after tamoxifen treatment. Gα_i2_-specific PCR products from each genotype were sequenced (Figure 3A). This analysis confirmed that the Gα_i2_ cDNA sequence from Gα_i2_^fl-G184S/fl-G184S^ mice prior to tamoxifen treatment was identical to the previously published mouse cDNA sequence [22,23]. In contrast, the Gα_i2_ cDNA sequence from Cre-ER^T2^/Gα_i2_^+/fl-G184S^, or Cre-ER^T2^/Gα_i2_^fl-G184S/fl-G184S^ mice revealed the nucleotide changes that were introduced to change the codon for glycine at 184 to serine. Peak heights from the chromatograms were used to quantify the relative amount of mutant mRNA expression in several tissues [24]. In mice that were homozygous for fl-G184S (Gα_i2_^fl-G184S/fl-G184S^), we found 60 – 100% conversion to the mutant sequence in most tissues evaluated (Figure 3B). As expected, heterozygous animals (Gα_i2_^+/fl-G184S^) exhibited lower rates of conversion because they retained one copy of the wildtype allele. The lack of conversion in brain was presumably due to lack of Cre-ER^T2^ expression in post-mitotic neurons, and low brain penetration by tamoxifen [20].

**Figure 4:**
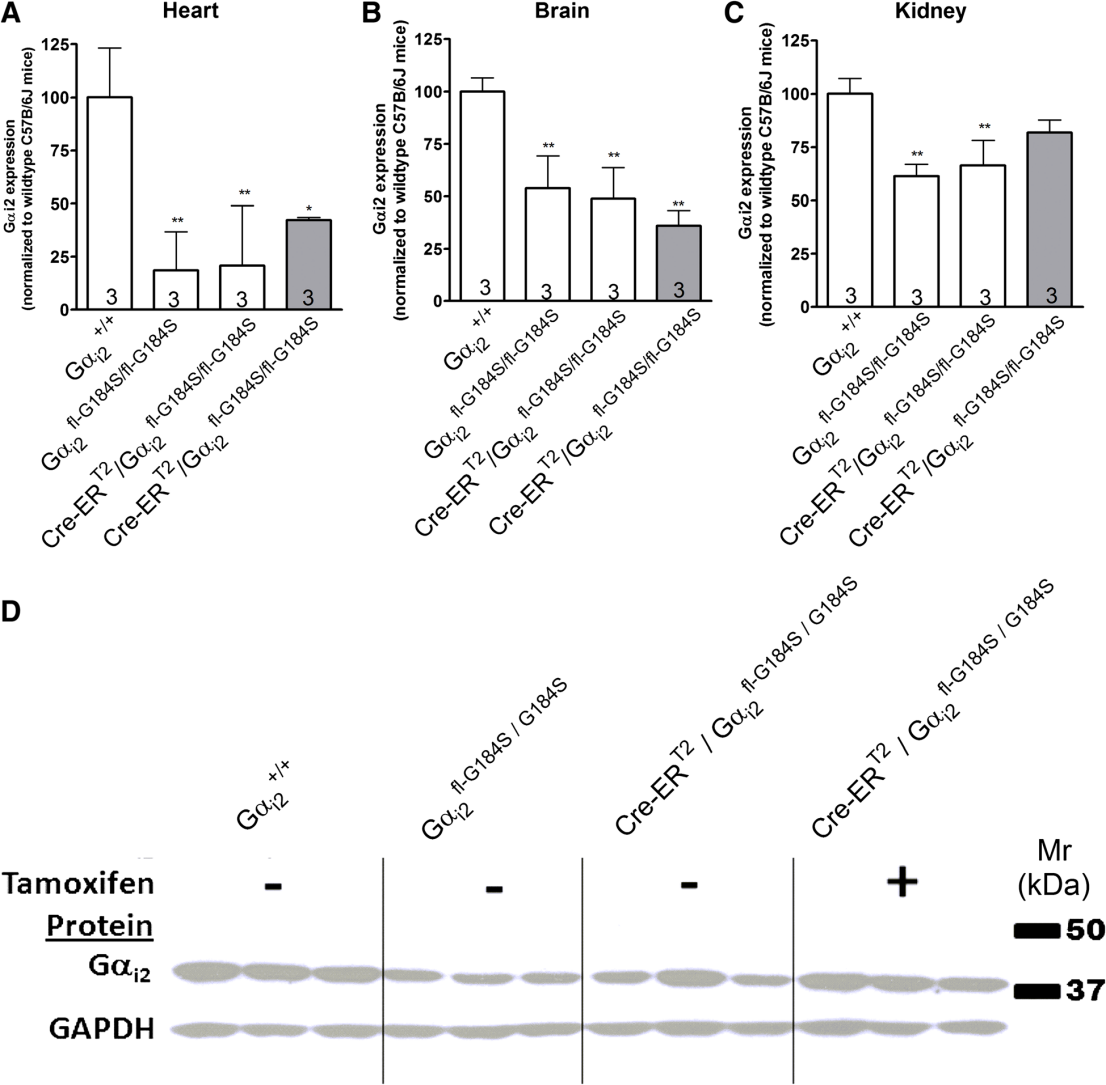
**Gα**_**i2 **_**protein expression is reduced in mice conditionally expressing Gα**_**i2**_^**G184S**^**.** Gα_i2_ protein expression was measured by Western blot in hearts **(A)**, brains **(B)**, and kidneys **(C)** isolated from mice treated with tamoxifen (50 mg/kg; shaded bars) or vehicle (nonshaded bars) for 5 consecutive days. The membrane was stripped and reblotted with a GAPDH antibody as a loading control. Values represent the mean ± SE of tissues from three different animals for each genotype. Data are normalized to Gα_i2_ protein expression in tissues from wildtype C57B/6J mice that do not express Cre and do not carry the Gα_i2_^G184S^ mutation. Panel **D** shows a representative blot of kidneys from three different animals of each genotype. * and ** indicate significant differences (p < 0.05 and p < 0.01, respectively) relative to control mice (G_αi2_^+/+^) that do not carry the Cre-ER^T2^ or G_αi2_^fl-G184S/fl-G184S^ genes.

Surprisingly, Gα_i2_ protein expression levels were significantly reduced in several organs (heart, brain, and kidney) isolated from Gα_i2_^fl-G184S/fl-G184S^ and Cre-ER^T2^/Gα_i2_^fl-G184S/fl-G184S^ mice compared to Gα_i2_^+/+^control animals that do not express Cre or the Gα_i2_^G184S^ mutation (Figure 4). We also assessed whether there were compensatory changes in other G proteins in heart ventricles. We found no detectable levels of Gα_o_, Gα_i1_, or Gα_i3_ in wildtype ventricles nor were they present in Gα_i2_^fl-G184S/fl-G184S^ or Cre-ER^T2^/Gα_i2_^fl-G184S/fl-G184S^ ventricles either before or after tamoxifen treatment (data not shown). This is consistent with previous reports that Gα_i2_ is a predominant Gα_i_ isoform in the myocardium [25,26].

**Figure 5:**
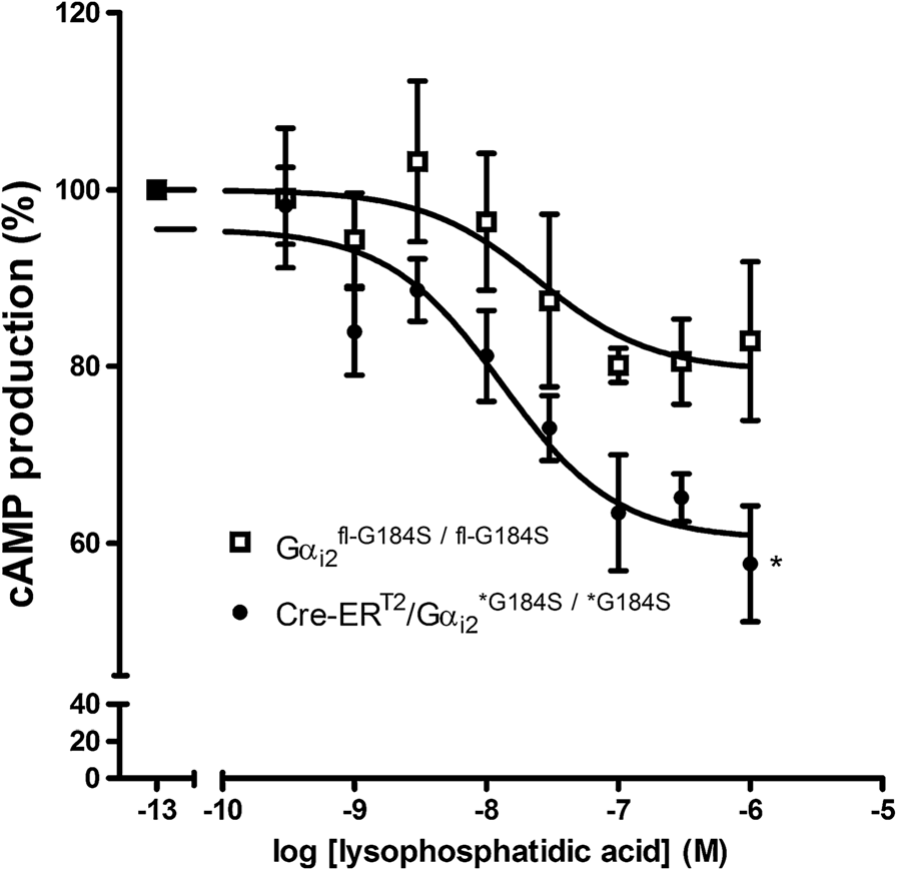
**Enhanced inhibition of forskolin-stimulated adenylate cyclase by lysophosphatidic acid following conditional induction of the Gα**_**i2**_^**G184S **^**mutation in adult cardiac fibroblasts.** Concentration-response curves were generated from adult cardiac fibroblasts isolated from three different mice of each genotype after treatment with tamoxifen (50 mg/kg i.p. on five consecutive days). Data from 5 independent determinations for both Gα_i2_^fl-G184S/fl-G184S^ and Cre-ER^T2^/Gα_i2_^*G184S/*G184S^ are shown as mean ± SE. Data are normalized to cAMP production in the presence of 10^-13^ M lysophosphatidic acid. Two way ANOVA revealed a significant effect for genotype [F = 5.58 (1,8) p < 0.05] and a significant effect for lysophosphatidic acid concentration [F = 12.09 (8, 64) p < 0.0001]. * indicates a significant difference (p < 0.05 compared to Gα_i2_^fl-G184S/fl-G184S^ cells treated with 10^-6^M LPA) using Bonferroni’s post test.

We previously demonstrated that embryonic fibroblasts from conventional (i.e. non-conditional) Gα_i2_^G184S^ knock-in mice have enhanced activity in several biochemical and physiological measures of the Gα_i_ signaling pathway [8]. Thus, we investigated the functional consequences of conditional Gα_i2_^G184S^ expression at the cellular level in a cAMP inhibition assay. Adult cardiac fibroblasts from mice treated with tamoxifen (which developed ~90% mutant allele expression, data not shown) were isolated and cultured. cAMP production in these cells was measured following exposure to forskolin (10 μM) and increasing concentrations of lysophosphatidic acid (LPA). Data were compared by two-way ANOVA with genotype and LPA concentration as variables. A significant effect of genotype (P < 0.05) in the inhibition of cAMP production was observed. Bonferroni’s post test revealed that ACF from the conditional knock-in mice (Cre-ER^T2^/Gα_i2_) had significantly (P < 0.05) augmented inhibition of cAMP production with the highest concentration of LPA compared to cells from Gα_i2_^fl-G184S/fl-G184S^ mice that do not convert to the G184S mutation (Figure 5). These data are consistent with our previous finding that LPA-induced inhibition of adenylate cyclase activity is enhanced in embryonic fibroblasts in the nonconditional Gα_i2_^G184S^ model [13], and they provide evidence that agonist-induced Gα_i2_ signaling is enhanced in cardiac fibroblasts that conditionally express the Gα_i2_^G184S^ allele.

**Figure 6:**
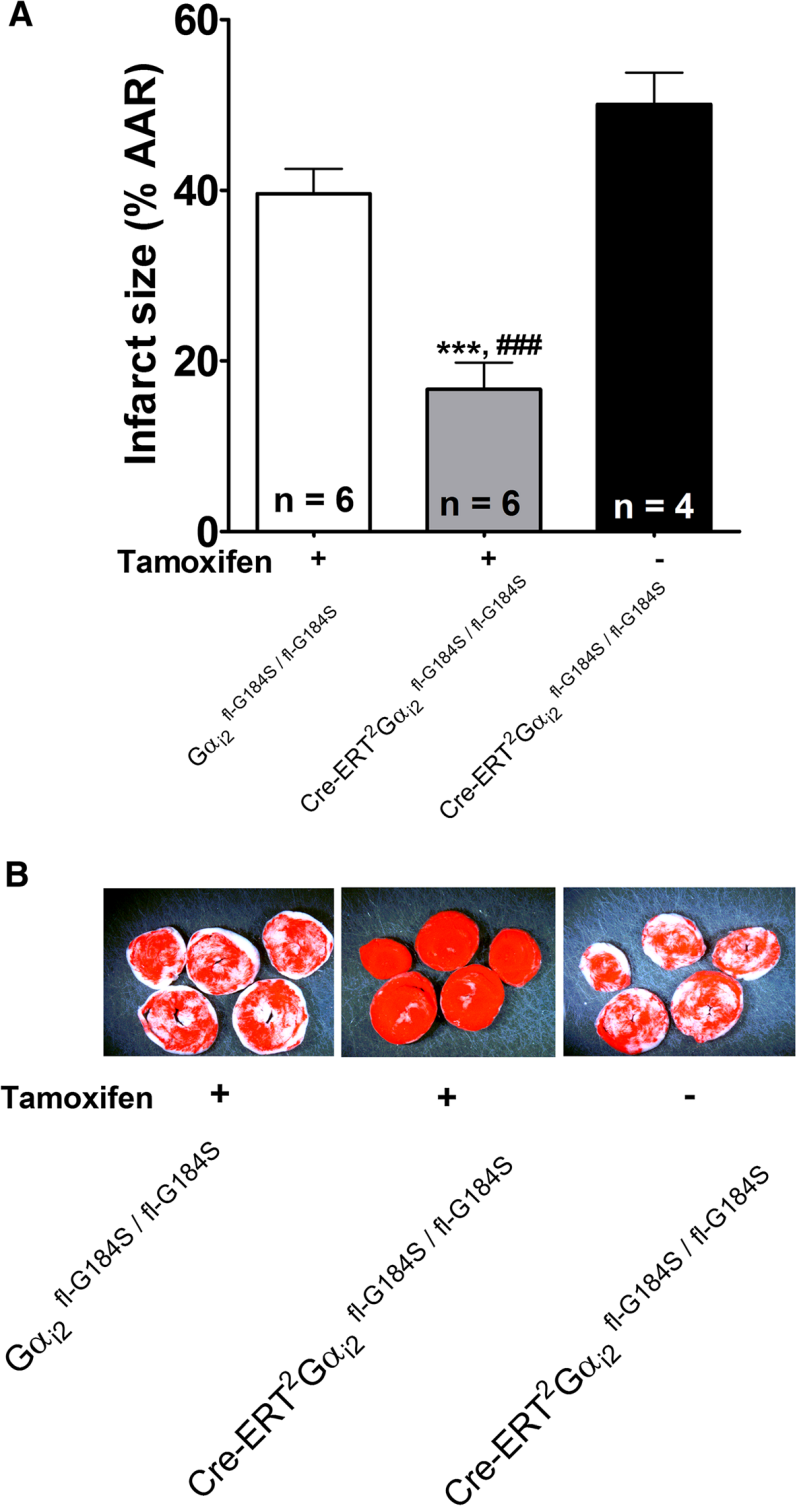
**Conditional expression of Gα**_**i2**_^**G184S**^**protects the heart from ischemic injury.** Conditional expression of Gα_i2_^G184S^ significantly (p < 0.001) decreased infarct sizes in hearts exposed to 30 min of ischemia **(A)**. Values represent means ± SE. The number of replicates for each group is shown within the bars. Data were compared by one way ANOVA and posthoc Tukey analysis. *** indicates a significant difference (p < 0.001) compared to hearts isolated from tamoxifen treated Gα_i2_^fl-G184S/fl-G184S^ mice, and ### indicates a significant difference (p < 0.001) compared to hearts isolated from Cre-ER^T2^/Gα_i2_^fl-G184S/fl-G184S^ mice that were treated with vehicle instead of tamoxifen. Photographs of representative triphenyltetrazolium chloride-stained hearts are shown in panel **B**.

We previously demonstrated that the conventional Gα_i2_^G184S^ mutant mice (both heterozygotes and homozygotes) are resistant to ischemia-reperfusion injury [13]. However, it was unclear whether this phenotype resulted from Gα_i2_^G184S^ expression during embryonic development or whether the cardioprotective phenotype requires chronic or acute Gα_i2_^G184S^ expression. In the present study, we found that ischemia-induced infarct size was significantly decreased in tamoxifen-treated Cre-ER^T2^/Gα_i2_^fl-G184S/fl-G184S^ compared to both vehicle-treated Cre-ER^T2^/Gα_i2_^fl-G184S/fl-G184S^ hearts and tamoxifen-treated Gα_i2_^fl-G184S/fl-G184S^ without Cre expression (Figure 6). Consistent with reduced myocardial infarct size (Figure 6), hearts isolated from tamoxifen-treated Cre-ER^T2^/Gα_i2_^fl-G184S/fl-G184S^ exhibited significantly enhanced postischemic recovery of developed pressure compared to hearts isolated from either vehicle-treated Cre-ER^T2^/Gα_i2_^fl-G184S/fl-G184S^ mice or tamoxifen-treated Gα_i2_^fl-G184S/fl-G184S^ mice that do not express Cre-ER^T2^ (Table 1). Postischemic recovery of + dP/dT and –dP/dT were also enhanced in hearts isolated from tamoxifen treated Cre-ER^T2^/Gα_i2_^fl-G184S/fl-G184S^ animals. Postischemic recovery of end diastolic pressure was nominally lower in tamoxifen-treated Cre-ER^T2^/Gα_i2_^fl-G184S/fl-G184S^ compared to the other groups (Table 1). However, this did not reach statistical significance. These data are consistent with our previous report that nonconditional expression of Gα_i2_^G184S^ protects the heart from ischemic injury [13]. Importantly, they extend our previous findings by demonstrating that the cardioprotective phenotype does not result from embryonic or chronic expression of Gα_i2_^G184S^. Rather, acute disruption of interactions between Gα_i2_ and endogenous RGS proteins is sufficient to induce cardioprotection.

**Table 1:** Preischemic and postischemic recovery of contractile function

	**Preischemic**			**Postischemic recovery**		
	**Gα**_**i2**_^**fl-G184S/fl-G184S**^ **+ tamoxifen (n = 6)**	**Cre-ER**^**T2**^**/Gα**_**i2**_^**fl-G184S/fl-G184S**^ **+ tamoxifen (n = 6)**	**Cre-ER**^**T2**^**/Gα**_**i2**_^**fl-G184S/fl-G184S**^ **+ vehicle (n = 4)**	**Gα**_**i2**_^**fl-G184S/fl-G184S**^ **+ tamoxifen (n = 6)**	**Cre-ER**^**T2**^**/Gα**_**i2**_**/**^**fl-G184S/fl-G184S**^ **+ tamoxifen (n = 6)**	**Cre-ER**^**T2**^**/Gα**_**i2**_^**fl-G184S/fl-G184S**^ **+ vehicle (n = 4)**
**Developed pressure (mmHg)**	130 ± 6	140 ± 8	132 ± 6	45 ± 5	78 ± 8 * #	29 ± 7
**+dP/dT (mmHg/sec)**	6375 ± 570	6145 ± 621	4587 ± 251	2223 ± 321	3557 ± 521 **#**	985 ± 258
**-dP/dT (mmHg/sec)**	-4471 ± 291	-4601 ± 275	-3837 ± 235	-1600 ± 197	-2581 ± 263 * #	-908 ± 243
**End diastolic pressure (mmHg)**	5.7 ± 1.0	6.2 ± 0.9	5.0 ± 1.0	30 ± 2	21 ± 3	33 ± 7
**Coronary flow rate (ml/min)**	4.5 ± 0.4	4.1 ± 0.3	4.6 ± 0.4	2.7 ± 0.3	2.6 ± 0.2	2.9 ± 0.4

Preischemic contractile function was measured prior to 30 minutes of ischemia. Postischemic recovery of contractile function was measured following 1 hour of reperfusion. Data from each group were compared by one way ANOVA and posthoc Tukey analysis (preischemic and postischemic values were analyzed separately from one another). There were no significant differences between groups for any parameters of preischemic contractile function. *indicates a significant difference (p < 0.05) in postischemic recovery of contractile function compared to hearts isolated from tamoxifen treated Gα_i2_^fl-G184S/fl-G184S^ mice. # indicates a significant difference (p < 0.01) in postischemic recovery of contractile function compared to hearts isolated from Cre-ER^T2^/Gα_i2_^fl-G184S/fl-G184S^ mice that were treated with vehicle instead of tamoxifen.

## Discussion

We previously described a mouse model in which signaling by Gα_i2_ was enhanced by introduction of a point mutation (G184S) that blocks negative regulation by RGS proteins [8]. These mice exhibited a number of complex and interesting phenotypes including protection from cardiac ischemia/reperfusion injury [13]. However, it was unclear whether this cardioprotective phenotype resulted from changes in Gα_i2_ signaling during embryonic development. It was also unknown whether chronic Gα_i2_^G184S^ expression was required or whether acute expression was sufficient to protect the heart from ischemic injury. These are important issues because the potential finding that embryonic or chronic expression of Gα_i2_^G184S^ is required for cardioprotection would likely preclude the use of pharmacological RGS inhibitors from therapeutic use in the treatment of ischemic heart disease. The present study extends our previous findings by demonstrating that acute (days rather than months) disruption of interactions between Gα_i2_ and endogenous RGS proteins is sufficient to protect the heart from ischemic injury. These data suggest that RGS proteins might provide novel therapeutic targets to protect the heart from ischemic injury.

Conditional expression of Gα_i2_^G184S^ resulted in a 55% reduction in infarct size relative to hearts isolated from tamoxifen-treated mice that did not express Cre-ER^T2^, and a 65% reduction in infarct size compared to hearts isolated from mice that expressed Cre-ER^T2^ but were not treated with tamoxifen. By comparison, we previously reported that nonconditional expression of Gα_i2_^G184S^ resulted in 41% and 68% reductions in infarct sizes in hearts isolated from mice that were heterozygous or homozygous for the Gα_i2_^G184S^ mutation, respectively [13]. Recovery of developed pressure, +dP/dT, and -dP/dT were significantly increased in both models of Gα_i2_^G184S^ expression (Table 1 and [13]). Thus, conditional expression of this mutation produced a cardioprotective phenotype that was similar to the phenotype previously observed in the conventional (nonconditional) Gα_i2_^G184S^ knockin model.

Mice expressing the Gα_i2_^G184S^ in a nonconditional fashion also exhibit several noncardiac phenotypic differences including: resistance to high fat diet-induced weight gain and resistance to the development of insulin insensitivity, reduced embryonic viability, elevated neutrophil counts, and antidepressant-like behavior [5,8,14]. We anticipate that the conditional Gα_i2_^G184S^ expression model described here will be helpful in characterizing the temporal requirements (acute versus chronic) of Gα_i2_^G184S^ expression for some of these phenotypes. Our finding that tamoxifen produced very little conversion to the Gα_i2_^G184S^ mutation in the brain suggests that this model may not be useful for characterizing the anxiolytic or antidepressant-like behavior that has been previously reported in nonconditional Gα_i2_^G184S^ mice [5]. On one hand, this represents a significant limitation of this model. However, the observation that the conventional Gα_i2_^G184S^ model, but not the conditional Gα_i2_^G184S^ model, expresses the G184S mutation in the brain might be useful for elucidating the ability of enhanced Gα_i2_ signaling in the central nervous system to impact the function of peripheral tissues. The reason for a lack of conversion to the Gα_i2_^G184S^ mutation in the brain is unclear. However, other investigators using the Cre-ER^T2^ system have reported similar results. Seibler et al. confirmed that Cre-ER^T2^ is expressed in the brain and suggested that the lack of recombination may reflect low local concentrations of 4-hydroxytamoxifen [27].

The tamoxifen- and Cre-dependent recombination and expression of mutant mRNA meets the criteria planned for the engineered Gnai2 locus: lack of mutant mRNA expression before tamoxifen treatment with good conversion to the G184S mutation after tamoxifen treatment. We did observe reduced Gα_i2_ protein expression in the heart, kidney, and brain of Gα_i2_^flG184S/fl-G184S^ and Cre-ER^T2^/Gα_i2_^flG184S/fl-G184S^ mice relative to wildtype mice that do not express Cre or carry the G184S mutation, suggesting that the loss of introns in the minigene that carries the wildtype sequence and/or the residual LoxP or FLP recombinase (FRT) sites may perturb regulatory and/or splicing elements. However, the observation that LPA-induced inhibition of cAMP production is augmented in cardiac fibroblasts isolated from Cre-ER^T2^/Gα_i2_^flG184S/fl-G184S^ mice (Figure 5) indicates that agonist-induced signaling via Gα_i2_ is actually increased (relative to fibroblasts that do not express the mutation), despite decreased Gα_i2_ expression levels. This presumably occurs because the negative regulatory effect of RGS proteins toward Gα_i2_ is disrupted in these cells. Many different Gα_i_-coupled receptors are known to protect the heart from ischemia/reperfusion injury. Thus, the finding that conditional expression of Gα_i2_^G184S^ produces a cardioprotective phenotype is also consistent with enhanced Gα_i2_ signaling despite decreased Gα_i2_ protein expression.

## Conclusions

In conclusion, the conditional Gα_i2_^G184S^ knock-in model described here permits the disruption of interactions between Gα_i2_ and endogenous RGS proteins in a time-dependent manner. Our data demonstrate that acute (rather than chronic) disruption of interactions between RGS proteins and Gα_i2_ is sufficient to protect the heart from ischemic injury. This suggests that the development of chemical inhibitors of RGS proteins or other agents that disrupt interactions between RGS proteins and Gα_i2_ might provide new therapeutic tools to protect the heart from ischemic injury. In addition, we anticipate that this transgenic mouse model will provide a valuable tool for characterizing the mechanisms by which disruption of RGS protein-Gα_i2_ interactions confers other phenotypes that have been identified in mice expressing Gα_i2_^G184S^.

## Abbreviations

ACF: Adult cardiac fibroblast; ES: Embryonic stem; Gα_i2_^+/+^: Wildtype Gα_i2_ allele; Gα_i2_^G184S^: Gα_i2_ allele with point mutation; PCR: Polymerase chain reaction; RGS: Regulator of G protein signaling.

## Competing interests

The authors have no financial or nonfinancial competing interests.

## Authors’ contributions

RRN, SP, and XH conceived and designed the experiments. SP, BRR, KK, RAC, SMW, and XH performed the experiments. SP, RRN, and BRR analyzed the data and wrote the manuscript. All authors read and approved the final manuscript.

## Pre-publication history

The pre-publication history for this paper can be accessed here:

http://www.biomedcentral.com/2050-6511/15/29/prepub
